# Our Medical College

**Published:** 1869-02

**Authors:** 


### OUR MEDICAL COLLEGE.

The 22d regular term of instruction in this Institution is now in progress. The number in attendance is larger than that of the last session, and in point of talent has not been surpassed by any former class. Material is abundant, and the means for clinical instruction, both medical and surgical, will compare favorably with the older institutions of the East.

In order that the practitioner and medical student may more fully appreciate our clinical advantages, we herewith append a list of cases operated upon and treated before the class of last session:

### MEDICAL CLINIQUES.

| | | | | | |
|---|---|---|---|---|---|
| Enuresis | 10 | Hemicrania | 4 | Anasarca | 3 |
| Sciatica | 3 | Neuralgia | 7 | Dilatation of Stomach | 2 |
| Leucorrhœa | 4 | Intermittent Fever | 7 | Dysentery (acute) | 2 |
| Phthisis | 6 | Remittent Fever | 5 | Dysentery (chronic) | 6 |
| Spinal Irritation | 3 | Hemiplegia | 4 | Chorea | 4 |
| Rheumatism | 4 | Paraplegia (incomplete) | 1 | Tonsillitis (acute) | 4 |
| Diarrhœa | 5 | Icterus | 4 | Dyspepsia | 3 |
| Chronic Laryngitis | 4 | Menorrhagia | 4 | | |
| Chronic Bronchitis | 3 | Ascites | 5 | Total | 112 |
| Acute Bronchitis | 5 | | | | |

SURGICAL CLINIQUES.

| | | | | | |
|---|---|---|---|---|---|
| Necrosis | 5 | Dropsy of Eye | 1 | Excision Tonsils | 2 |
| Artificial Anus | 2 | Double Entropion | 1 | Strumous Ulcers | 2 |
| Amputations | 4 | Conjunctival Adhesion | 3 | Nasal Polypus | 2 |
| Hare Lip | 4 | Evulsion Cilia | 3 | Synovitis (acute) | 1 |
| Fractures | 5 | Pterygium | 2 | Synovitis (chronic) | 2 |
| Punctured Fracture of | | Otorrhœa | 8 | Cheiloplastic operation | 2 |
| Cranium | 1 | Caries | 6 | Ligature Hemorrhoids | 1 |
| Opthalmia (strumous) | 5 | Concussion of Brain | 1 | Talipes Varus | 3 |
| Blepharoplasty | 1 | Compression of Brain | 1 | Enchondroma | 2 |
| Conjunctivitis | 10 | Trephining | 1 | Facial Deformity | 1 |
| Granular Lids | 7 | Lithotomy (bilateral op- | | Osteitis | 1 |
| Corneal Opacity | 8 | eration | 2 | Insertion Seton | 2 |
| Entropion | 4 | Incised Wounds | 6 | Hypertrophied Tonsils | 1 |
| Strabismus | 6 | Hydrocele | 1 | | |
| Cataract | 4 | Dislocated Lens | 1 | Total | 126 |

